# Dynamic of Covid-19 representations: time, cultural and social factors

**DOI:** 10.1192/j.eurpsy.2023.500

**Published:** 2023-07-19

**Authors:** E. R. Semenova, O. Mitina, E. Pervichko, J. Konyukhovskaya, O. Stepanova

**Affiliations:** Moscow State University named after ‘M. V. Lomonosov’, Moscow, Russian Federation

## Abstract

**Introduction:**

We have already noted in our studies (Pervichko et. al., 2020, 2021) that collective representations about Covid-19 disease, based on its totality, the catholicity of its impact on all spheres of people’s lives, are socially and culturally conditioned: they have their own specifics in different countries, associated with many social, economic, political factors. Moreover, these perceptions change over time.

**Objectives:**

To identify the differences in the Covid-19 representations in residents of different countries and at different stages of the pandemic.

**Methods:**

Modified questionnaire of the internal picture of the disease (Broadbent, 2006), consisting of 8 questions about COVID-19 pandemic. All the items were rated using a 0-to-10 response scale. 1-5 items assess cognitive illness representations. Items 6 and 8 assess emotional representations. Item 7 assesses illness comprehensibility.

**Results:**

To test of the research hypothesis, we used the data accumulated on our platform since April 2020. We selected subsamples: Russian respondents who took the survey in April-May 2020 (1st wave) (1), June-September 2020 (decline) (2), October - December 2020 (3) (2nd wave), 1st half of 2021 (4). At the same time as sample 4, respondents from Azerbaijan (5) and Uzbekistan (6) were surveyed. Finally, there was another sample of Uzbekistan respondents surveyed in the 1st half of 2022 (7). Respondents of both sexes and different ages participated in each sample, with a total of 2908 people. The table 1 shows the sample means for all items in all samples.

In all seven samples, women are characterized by responses indicating their greater tension, stress, and psychological fatigue caused by life in a pandemic. In almost all samples, younger respondents demonstrate greater involvement in the disease and its experiences. Perhaps this is due to the fact that social restrictions affected young respondents to a greater extent.

It was shown that with each new wave, the indicators for the 2, 5, 8 grow, and 3, 4, 7 decreases. At the same time, since no one canceled the pandemic, this year, along with a decrease in indicators for all items corresponding to stress and anxiety, ideas about the possible duration of the pandemic are growing.

**Image:**

**Figure:**
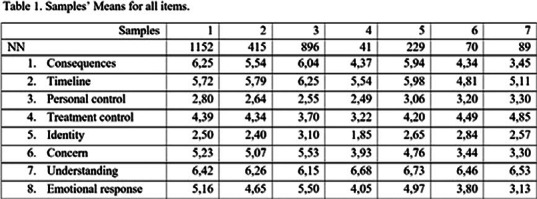

**Conclusions:**

We can say that people have adapted to coexist with this disease and are ready for its continued presence in the world. The average indicators of Azerbaijani respondents indicate a more tense attitude towards the pandemic compared to Russia and Uzbekistan. It can be assumed that this is due to longer and more extensive state anti-epidemiological measures in Azerbaijan.

**Disclosure of Interest:**

None Declared

